# Illumination systems in vitreoretinal surgery: a comparative review of safety and efficacy

**DOI:** 10.1186/s40942-026-00918-y

**Published:** 2026-08-05

**Authors:** Massimo Lupo, Sergio Lebet, Andrea Rescalli, Simone Micalizzi, Andrea Govetto, Paolo Oliva, Mario R. Romano

**Affiliations:** 1 Humanitas University, Via Rita Levi Montalcini 4, Pieve Emanuele - Milan, 20072 Italy; 2 IRCCS Humanitas Research Hospital, Via Alessandro Manzoni, 56, Rozzano, Milano 20089 Italy; 3 Department of Biomedical Sciences, Humanitas University, Pieve Emanuele-Milan, Italy

**Keywords:** Endoillumination, Vitreoretinal surgery, Chandelier illumination, Retinal phototoxicity

## Abstract

**Background:**

Endoillumination is essential in modern vitreoretinal surgery, enabling visualization during peripheral vitreous shaving, retinal detachment repair, and other bimanual maneuvers. Chandelier illumination provides a stable wide-field light source through a separate port and should be regarded as an adjunct to conventional handheld fiber-optic endoillumination rather than as a replacement. This narrative review compares handheld, chandelier, and emerging LED-based illumination systems, focusing on surgical utility, visualization, illumination geometry, and retinal light-exposure considerations.

**Main text:**

A PubMed literature search was conducted for studies published between 1990 and April 2026. After screening 401 records and evaluating 72 full-text articles, 24 studies were included in the qualitative synthesis. These comprised in vitro investigations on retinal pigment epithelial cells, ex vivo porcine-eye irradiance measurements, prospective clinical series, and one randomized controlled trial. Across the available evidence, the most consistent safety advantage of chandelier illumination was related to delivery geometry. By increasing the working distance from the retina, chandelier systems markedly prolong retinal threshold exposure time compared with handheld endoillumination probes, largely in accordance with the inverse-square law. LED-based systems demonstrated variable safety profiles depending on spectral output, with some devices showing prominent short-wavelength peaks and others exhibiting lower blue-light fractions when compared to xenon sources. Spectral filtering, particularly amber filtering during dye-assisted procedures, further reduced photochemical hazard. Three-dimensional digital visualization systems also reduce the level of physical intraocular illumination required from conventional lighting systems while maintaining adequate surgical visualization. Additional emerging technologies, including micro-LED chip-on-tip probes, transscleral illumination, omnidirectional diffusers, and portable LED devices, suggest a broader transition toward fiber-free or fiber-optimized illumination platforms.

**Conclusions:**

Chandelier-based LED illumination systems appear to offer a versatile and generally safer addition to traditional wired endoilluminators. Their clinical value lies in improving peripheral visualization and facilitating complex bimanual maneuvers. Safety advantages are mainly related to illumination geometry, working distance, light distribution, and reduced need for repeated handheld probe manipulation. Nevertheless, retinal safety depends on the combined effects of emission spectrum, delivery geometry, intraocular distance, exposure duration, spectral filtering, surgical technique, and visualization system.

**Supplementary Information:**

The online version contains supplementary material available at https://doi.org/10.1186/s40942-026-00918-y.

## Introduction

Vitreoretinal surgery has undergone profound technological evolution over the last few decades, with major advances in instrumentation, fluidics, visualization platforms, and intraocular illumination [1, 2]. Among these developments, endoillumination has become a critical component of modern vitreoretinal surgery by enabling direct visualization of transparent and semi-transparent intraocular tissues while minimizing the reflections and light scattering associated with microscope-based coaxial illumination. These improvements have been particularly relevant for the management of complex procedures, including membrane dissection, peripheral vitreous shaving, retinal detachment repair, and bimanual surgery [1, 2].

**Fig. 1:**
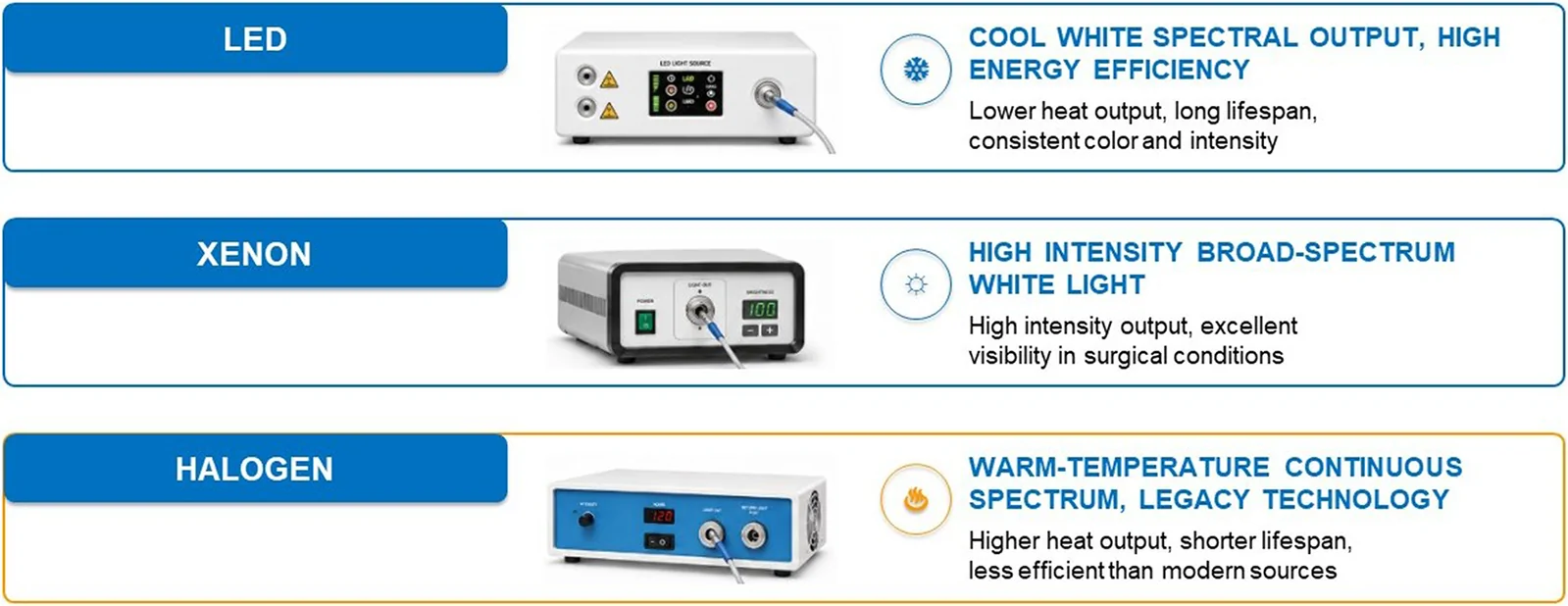
Comparison of ophthalmic endoillumination light sources. All light sources deliver illumination via fiber-optic systems for endoillumination during ophthalmic surgery

Historically, vitreoretinal surgery relied on fiberoptic illumination systems coupled with halogen and metal halide light sources [2, 3]. Although these systems represented a crucial step, their luminous output became increasingly inadequate with the transition toward smaller-gauge instrumentation, given that the reduction in fiber diameter lowers the amount of light that could be transmitted to conventional fiber-optic systems. This limitation stimulated the adoption of more powerful illumination technologies, particularly xenon, mercury vapors, and, more recently, light-emitting diode (LED) systems, which offer greater brightness and improved durability [4–8]. Figure 1 shows a comparison of ophthalmic endoillumination light sources.

In parallel, the progressive refinement of endoilluminator design and light delivery has expanded the surgeon’s ability to operate safely in both the posterior pole and the peripheral retina [9, 10].

**Fig. 2:**
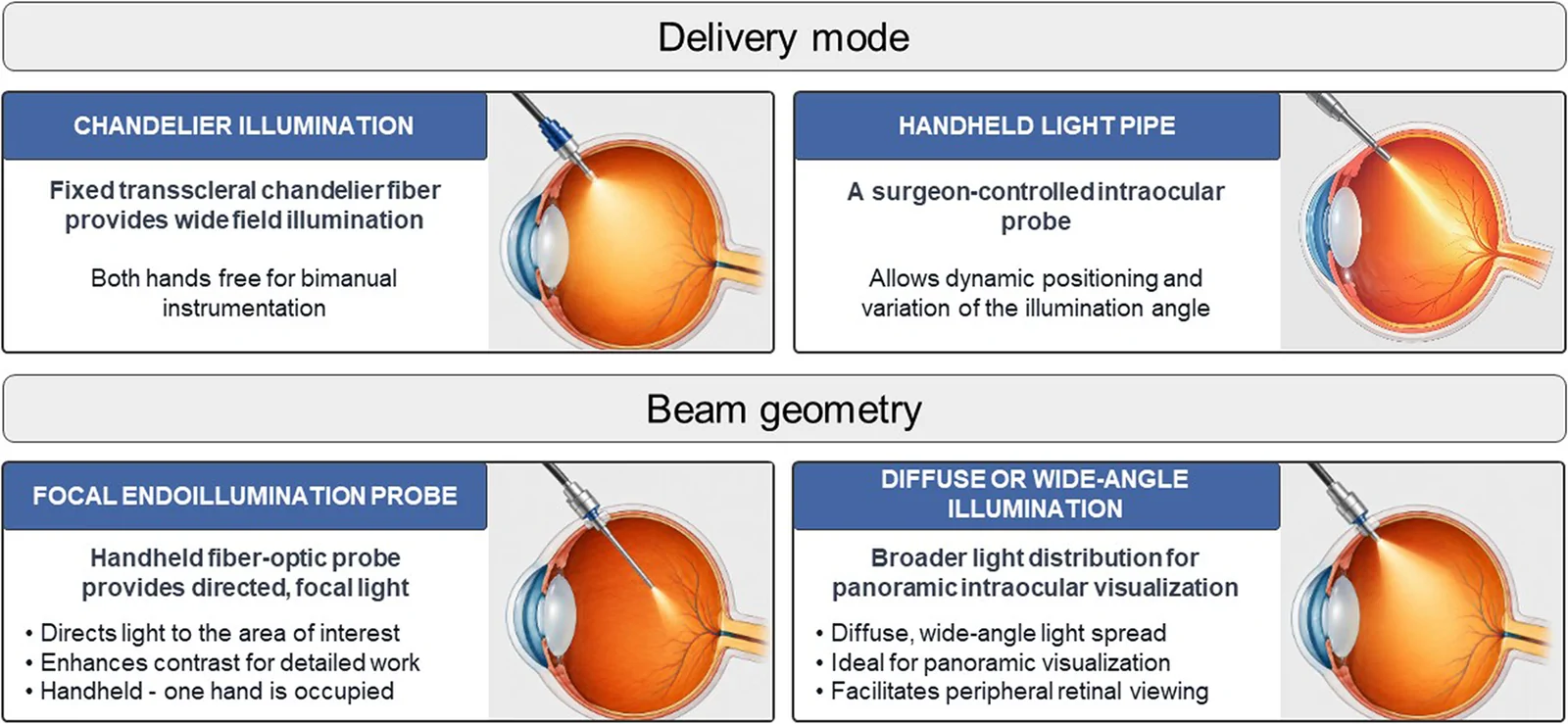
Classification of vitreoretinal endoillumination according to delivery mode and beam geometry

Chandelier illumination systems represented another important innovation, providing diffuse intraocular illumination through a separate port and thereby freeing both hands for surgical manipulation since their introduction by Eckardt et al. in 2003 [11]. At the same time, advances in visualization technology, including wide-angle viewing systems and 3D digital visualization system, have further enhanced the ability to perform vitreoretinal surgery under lower illumination settings while preserving adequate image quality and depth perception [2, 9]. Figure 2 summarizes the main endoillumination techniques adopted in vitreoretinal surgery.

Despite the improvements in surgical visualization, retinal safety remains a central concern [1, 8, 10]. Unlike physiologic light entering through the anterior segment, endoillumination exposes the retina directly to high-intensity intraocular light, thereby increasing the potential for light-induced injury [3, 5, 6, 12]. Multiple factors, including wavelength, light intensity, exposure duration, working distance, and the optical characteristics of the illumination system, influence phototoxicity during vitreoretinal surgery [1, 9]. Shorter wavelengths, especially in the blue spectrum, are considered particularly relevant in photochemical retinal injury, and the risk may be further modified by intraoperative conditions and by the interaction between light sources, filters, dyes, and ocular tissues [1, 10].

Several strategies have been introduced to mitigate retinal light exposure, including spectral filtering, increased working distance, improved probe design, and newer light sources such as LEDs, which offer practical advantages, including reduced ultraviolet emission, lower heat generation, and adjustable spectral output [1, 6, 8, 13]. Experimental evidence has suggested that, under controlled conditions, both xenon and LED endoillumination can be used without inducing short-term retinal toxicity, although safety remains dependent on exposure parameters and cannot be considered independently of surgical context [1, 9].

Given the diversification of vitreoretinal illumination systems, a clearer comparison of their surgical utility, illumination geometry, and safety considerations is needed. Therefore, this narrative review aims to compare chandelier and conventional handheld fiber-optic endoillumination systems in vitreoretinal surgery, with specific focus on surgical utility, bimanual feasibility, illumination geometry, visualization, and retinal light exposure.

## Methods

### Search strategy

The key words used for the literature search were “vitreoretinal surgery”, “vitrectomy”, “pars plana vitrectomy”, “endoillumination”, “endoilluminator”, “fiberoptic technology”, “light pipe”, “chandelier illumination”, “intraocular illumination”, “LED”, “xenon”, “halogen”, “mercury vapor”, “luminosity”, “luminous flux”, “irradiance”, “phototoxicity”, “light toxicity”, “thermal injury”, “photochemical damage”, “blue light hazard”, “retinal damage”, “RPE damage”, “safety”, and “complications”, with the Boolean operators “AND” and “OR”, for exploring PubMed database.

We included all conceivable spelling variations of the key words, as well as their singular and plural forms, and incorporated all entry MeSH term variations. Their related MeSH expressions were also cross-checked to ensure a comprehensive search.

The literature search considered studies published between 1990 and April 2026 to ensure the inclusion of studies reflecting contemporary clinical practices and workplace environments. This period was chosen as it encompasses major shifts in illumination techniques.

### Eligibility criteria

Our research focused on the assessment of efficacy and safety between different illumination modalities used in vitreoretinal surgery. Studies were included in this narrative review if they met the following inclusion criteria: (1) involved patients undergoing vitreoretinal surgery (e.g., pars plana vitrectomy, retinal detachment repair, membrane peeling) or utilized an ex vivo/in vitro bench-top model simulating these conditions; (2) evaluated at least one intraocular endoillumination system, including handheld endoilluminators, chandeliers, or specific light sources (xenon, halogen, mercury vapor, LED, or hybrid); (3) reported quantifiable outcomes regarding efficacy (e.g., luminous flux, irradiance, field coverage, color rendering index), safety (e.g., phototoxicity, retinal light toxicity, thermal damage, or ANSI/ISO compliance), or surgical performance (e.g., operative time, bimanual feasibility, or surgeon ergonomics); and (4) utilized an RCT, prospective/retrospective cohort, observational, or experimental study design.

Studies were excluded based on the following criteria: (1) focus on non-relevant surgical fields (e.g., cataract, Laser-Assisted in Situ Keratomileusis (LASIK)) without applicable data for vitreoretinal illumination; (2) use of external, slit-lamp, or diagnostic illumination only; (3) conference abstracts without available full texts and pre-prints; (4) animal studies, unless specifically investigating retinal safety or phototoxicity thresholds directly informing human surgical safety; and (5) datasets that were duplicates, overlapping, or contained non-separable mixed data where vitreoretinal-specific outcomes could not be isolated. We restricted our search to the English, Italian, Spanish and French languages.

### Study selection

Two independent reviewers (M.L., S.L.) conducted the study selection process. During the first-level screening, titles and abstracts were assessed for eligibility, followed by a second-level full-text evaluation. Discrepancies between reviewers occurred in fewer than 1% of the screened records and were all resolved through discussion, without the need for a third reviewer. Study inclusion was based on their relevance to the research question and adherence to the predefined eligibility criteria. Reference list scanning was undertaken, as the initial assessment indicated that most citations within the identified sources were published within the defined time of this review.

A structured, qualitative critical appraisal of the included studies was performed to assess methodological robustness and clinical relevance. Given the narrative design and heterogeneity of the included clinical, laboratory, technical, and review articles, a formal, quantitative risk-of-bias assessment using standardized tools was considered not appropriate.

Instead, the appraisal approach was informed by established methodological frameworks, including the Scale for the Assessment of Narrative Review Articles (SANRA) scale for narrative reviews [14] and selected domains from the Joanna Briggs Institute critical appraisal tools [15], Animal Research: Reporting of In Vivo Experiments (ARRIVE) guidelines [16], and ToxRTool [17].

Studies were evaluated across domains reflecting internal validity, external validity/generalizability, reproducibility, and translational relevance. Internal validity was assessed in relation to the appropriateness of the study design, control of illumination exposure, validity of outcome measurements, and consistency between methods and conclusions. External validity and generalizability were assessed according to the extent to which the experimental model, illumination conditions, and surgical context reflected real-world vitreoretinal surgery. Reproducibility was judged based on the completeness of reporting of key technical parameters, including light source type, spectral output, illumination intensity, probe geometry, working distance, exposure time, and outcome assessment methods. Translational relevance was assessed by considering the applicability of the findings to clinical vitreoretinal surgery.

Each study was qualitatively categorized as having low, moderate, or high methodological concern, based on the assessed risk of bias and the extent to which methodological limitations could affect internal validity, external validity, reproducibility, and clinical applicability.

## Results

### Study selection

**Fig. 3:**
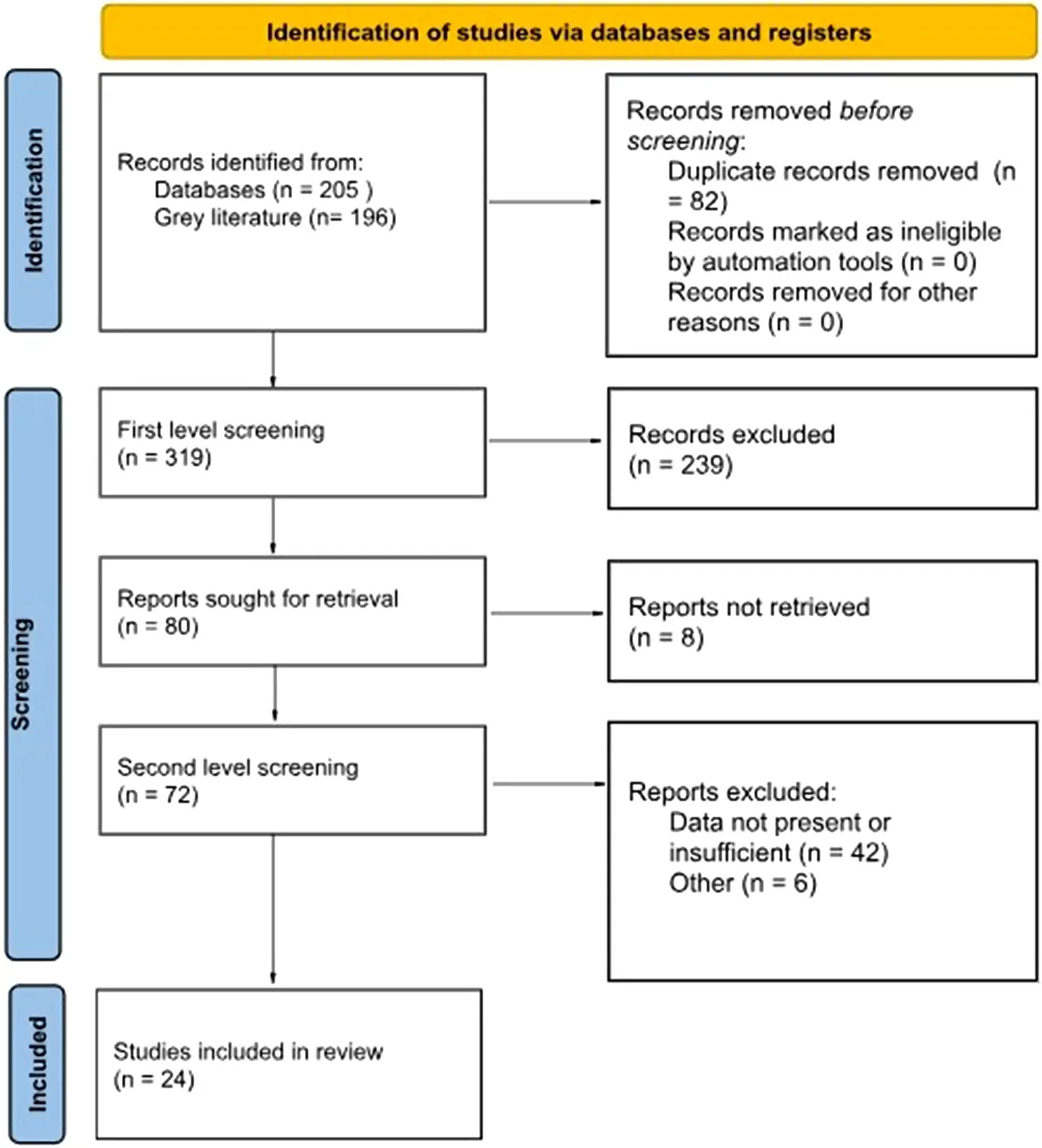
PRISMA 2020 flow diagram for new systematic reviews which included searches of databases and registers only

The search identified a total of 401 references. After removing 82 duplicates, 319 records remained for title and abstract screening. Of these, 239 were excluded as they were not relevant to the research question, leaving 80 articles for full-text retrieval. Eight articles could not be retrieved, resulting in 72 studies assessed for full-text eligibility. Following detailed evaluation, 48 studies were excluded. The most common reason for exclusion was that the research question of the paper did not provide relevant insights for this review. Ultimately, 24 studies were included in the narrative synthesis. Figure 3 summarizes the whole selection and inclusion process.

### Characteristics of included studies

The 24 sources encompassed a range of study designs, including in vitro laboratory investigations, clinical case series, prospective randomized trials, and narrative/literature reviews. The majority evaluated safety parameters (irradiance, phototoxicity thresholds, spectral output) of various endoillumination technologies, while a smaller subset assessed clinical efficacy outcomes.

A small fraction of the articles investigated new technologies. Supplementary Table 1 summarizes the key characteristics of each included study.

Full texts were retrieved for all 24 sources, which span over three decades (1993–2025) and reflect the substantial evolution of endoillumination technology. The 24 included studies comprised five narrative or expert reviews [3–5, 13, 18], six in vitro or ex vivo laboratory investigations on porcine eyes or retinal pigment epithelium (RPE) cell cultures [6, 12, 19, 20–22], three bench-test analyses of optical and physical parameters [20, 25, 26], one prospective randomized controlled trial [24], three clinical case series [25–27], and six reports on novel illumination devices or prototype systems [28–33].

### Critical appraisal of included studies

A structured qualitative critical appraisal of the included studies is reported in Supplementary Table 1. Overall, most studies were judged to have moderate methodological or translational concerns. Narrative reviews and early-stage device reports were generally associated with greater concerns regarding external validity and generalizability, mainly because of limited quantitative data, lack of standardized outcome measures, and limited clinical validation.

Experimental studies generally demonstrated stronger internal validity, with good control of illumination parameters and measurement conditions. However, their external validity was often limited by their predominantly in vitro, ex vivo, or animal-based design, which may not fully reproduce the optical, anatomical, and surgical conditions of human vitreoretinal surgery.

Clinical studies provided higher translational relevance and greater direct applicability to surgical practice, but were often constrained by small sample sizes, heterogeneous surgical indications, and limited long-term safety assessment. Technical and prototype-based studies contributed valuable information on device performance and illumination efficiency, but showed moderate concern because many lacked extensive clinical validation or standardized comparative outcomes.

### Synthesis of findings

The synthesis of the findings is reported as follows, divided into four domains for clarity. These domains address: the safety profiles across different illumination technologies, the clinical efficacy of reduced-illumination strategies, the effects of instrument design and gauge, and the role of spectral filters. For clarity, Table 1 summarizes the radiometric and photometric quantities used in this review.

**Table 1:** Radiometric and photometric quantities used in this review

Quantity	Symbol	Unit	Definition
Radiant power	Φ_e_	W	Total optical power emitted by the source
Irradiance	E_e_	W/cm^2^	Instantaneous radiant power incident per unit area
Radiant exposure (dose)	H_e_	J/cm^2^	Cumulative energy per unit area over a defined exposure time
Luminous flux	Φ_v_	lm	Light output weighted by the photopic response of the human eye
Illuminance	E_v_	lx	Luminous flux incident per unit area

#### Safety profiles across illumination technologies

##### Spectral output and blue light hazard

The spectral composition of light, particularly the proportion of short-wavelength (blue) emission, is the major determinant of photochemical retinal hazard. The studies consistently identify blue light (wavelengths ≤ 500 nm) as the most phototoxic component. However, safety cannot be ranked by light-source type alone, because it also depends on irradiance, intensity setting, probe geometry, working distance, exposure duration, and filtering.

**Table 2:** Spectral output and blue light hazard

Light source	Blue light proportion / Spectral peak	Reported safety-relevant findings
Halogen	Peak around 650 nm [19,22]; low short-wavelength component [6]	Lowest calculated hazard in Fehler et al. [6], but greater RPE toxicity than xenon/LED at equal lumen output in Sezer et al. [19]
Metal halide	Spectrum 300–800 nm; variable according to filter configuration [21]	Short calculated threshold times; selected 20G systems reduced A2E-laden RPE viability, while 25G systems did not [21]
Xenon, unfiltered	Blue proportion ~ 30.8% [7]; peak around 450 nm [19]	High luminous output and relevant short-wavelength component; safety depends on intensity, filtering, distance, and exposure time [6,7,22]
Xenon with 420–435 nm filter	Blue proportion ~ 28.8% [7]; bandpass ranges of 420–650 nm and 435–700 nm [22]	Filtering reduces selected short wavelengths, but safety remains dependent on irradiance, distance, and exposure duration [7,22]
Mercury vapor	Blue proportion ~ 14.1% [7]	Favorable blue-light proportion in Dithmar et al.; interpretation remains device-specific [7]
LED, white cool	Peaks around 455 and 565 nm; blue proportion ~ 22.3% [7]	Device-specific: ocuLED showed relatively low blue proportion [7], whereas LEDStar had a prominent ~ 440 nm peak and shorter calculated exposure times [6]
LED, white neutral	Peaks around 455 and 565 nm; blue proportion ~ 15.5% [7]	Lower blue proportion than cool white LED in Dithmar et al.; LED safety cannot be generalized across devices [6,7]

As shown in Table 2, mercury vapor sources consistently demonstrated favorable spectral safety profile among high-output light sources, with spectral peaks confined to the 550–580 nm range and the lowest blue light proportion (14.1%) [4, 7]. Xenon sources, while offering high luminous output, carried the highest blue light proportions (up to 30.8% unfiltered) [7], necessitating bandpass filters. The available data do not support a single safety ranking for LED illumination: while the Geuder ocuLED neutral white had a blue proportion of only 15.5% [7], the DORC LEDStar exhibited a pronounced emission peak around 440 nm that resulted in shorter maximum exposure times than either halogen or xenon [6]. This highlights that “LED” is not a monolithic category, but spectral characteristics vary substantially by manufacturer and color temperature selection.

##### Irradiance measurements and intraocular enhancement

A critical finding from Koelbl et al. (2019) [12] is that intraocular irradiance (mW/cm²) at the posterior pole was consistently 30–60% higher than the corresponding standardized reference measurement (flat detector in aqueous solution), depending on probe-to-retina distance. When integrated over a standardized 1-hour exposure, the resulting radiant exposure (J/cm²) exceeded the 10 J/cm² guide value defined by ISO 15004-2/ISO 15,752, above which irreversible retinal damage may occur. This discrepancy arises from the spherical geometry of the eye, which concentrates and reflects light onto the retina in ways not captured by standard flat-surface measurements. This finding has direct implications for interpreting all safety data derived from setups, suggesting that manufacturer-specified safe exposure times may be systematically overestimated.

**Table 3:** Irradiance measurements

System / Configuration	Irradiance at 5 mm (mW/cm²)	Irradiance at 10 mm (mW/cm²)
ocuLED cool white (LED) [7]	58	15
ocuLED neutral white (LED) [7]	58	15
Xenon (unfiltered) [7]	140	35
Xenon (435 nm filter) [7]	357	89
Mercury vapor (Photon II) [7]	162	41
Multiport chandelier (full intensity, 16.5 mm) [23]	56 (actual surgical setting)	Not measured at 10 mm
Multiport chandelier (50% intensity) [23]	3.7	Not measured
Conventional light pipes (at 5 mm) [23]	94–256	Not reported
Spotlight 23G (Alcon, intraocular) [12]	106.87 at 2 mm	7.24
Wide-angle 23G (Geuder, intraocular) [12]	30.26 at 2 mm	3.51
Fiberoptic-free microscope system [27]	30 (on retina)	Not applicable

**Fig. 4:**
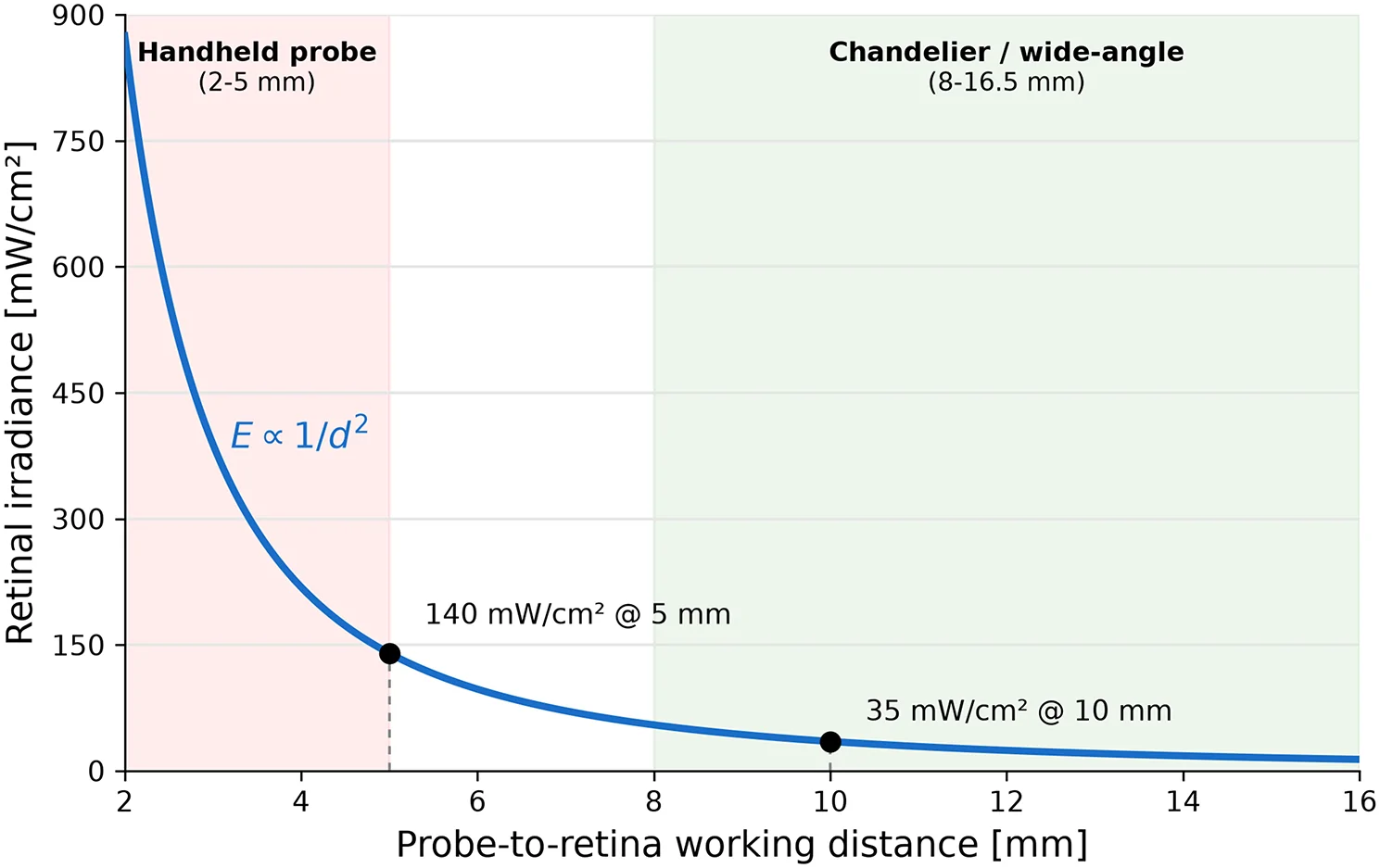
Inverse-square relationship between probe-to-retina working distance and retinal irradiance. Retinal irradiance decreases with the square of the working distance (E ∝ 1/d²). Filled circles show measured values for an unfiltered xenon source (140 mW/cm² at 5 mm and 35 mW/cm² at 10 mm), taken from Table 3. Shaded regions indicate the typical operating distances reported across the included studies for handheld probes (2–5 mm) and chandelier/wide-angle systems (8–16.5 mm)

The paired 5 mm and 10 mm measurements for each light source reported in Table 3 follow the inverse-square law, whereby retinal irradiance is inversely proportional to the square of the working distance (Fig. 4). For unfiltered xenon, for example, irradiance fell from 140 to 35 mW/cm² between 5 and 10 mm (i.e., a fourfold reduction) [7]. In clinical terms, this geometry explains why wide-angle endoilluminators and chandelier systems, which inherently operate at greater distances from the retina (typically 8–16.5 mm) [4, 23], deliver substantially lower retinal irradiance than handheld spotlight probes positioned at 2–5 mm [12]. Because the maximum tolerable exposure time is, for a given source, inversely proportional to irradiance, the same delivery geometry that lowers retinal irradiance also extends the safe exposure window, as detailed in Sect.  3.4.1.3.

##### Maximum tolerable exposure times

Maximum exposure times before exceeding photochemical safety thresholds (typically ICNIRP guidelines) [34] varied dramatically across light sources, distances, and filter configurations.

**Table 4:** Maximum tolerable exposure times

Light source / Configuration	Distance (mm)	Max exposure time
Halogen (hand-held, no filter) [6]	2	11.13 min
Halogen (hand-held, no filter) [6]	10	195.36 min
Xenon (hand-held, no filter) [6]	2	5.27 min
Xenon (hand-held, no filter) [6]	10	45.06 min
LED (DORC LEDStar, hand-held) [6]	2	2.09 min
LED (DORC LEDStar, hand-held) [6]	10	15.51 min
Metal halide (Brand 1, no filter) [8]	~ 5	0.2 min
Halogen (Brand 3, no filter) [8]	~ 5	2.1 min
With GG475 filter + 10 mm distance [8]	10	Up to 13 min
Alcon AHBI xenon (420 nm filter, 8 lm) [3]	4	7 min 43 s
Alcon Constellation xenon (435 nm filter, 8 lm) [3]	4	12 min 25 s
Mercury vapour (Stellaris PC/Photon 2, 8 lm) [3]	4	15 min 16 s
Alcon Accurus halogen (8 lm) [3]	8	33 min 48 s
Alcon Constellation xenon (8 lm) [3]	8	41 min 29 s
Mercury vapour (Stellaris PC/Photon 2, 8 lm) [3]	8	50 min 59 s
Synergetics 25G Awh Chandelier (max) [3]	~ 8	4 h 17 min 39 s
Multiport chandelier (halogen) [23]	16.5	110–270 min
Fibreoptic-free microscope system [27]	~ 25	111 min

As shown in Table 4, the most striking finding across multiple studies is the dramatically extended safe exposure times achievable with chandelier systems, such as the Synergetics 25G Awh chandelier, which at maximum illumination permitted over 4 h of use [3]. This advantage derives primarily from the greater and fixed working distance of chandelier systems [5, 23]. Increasing the distance from 4 mm to 8 mm alone increases retinal threshold time more than threefold [4, 5], close to the theoretical fourfold increase predicted by the inverse-square law.

##### In vitro phototoxicity on RPE cells

Table 5 reports the main findings on in vitro phototoxicity on RPE cells. In the study by Sezer et al., halogen illumination produced greater RPE cell toxicity than xenon or LED at comparable lumen output, while xenon and LED did not differ significantly from controls under the tested exposure conditions [19]. This contrasts with the theoretical hazard calculations that rank LED highest in photochemical risk due to blue spectral peaks [6]. The discrepancy likely reflects the role of bandpass filters in clinical xenon systems and the overall spectral integration rather than peak wavelength alone. The 25G systems consistently showed less phototoxicity than 20G systems, attributable to their lower total irradiance (26–32% less) [21].

**Table 5:** In vitro phototoxicity on RPE cells

Study	Light sources compared	Exposure conditions	Key cell viability findings
Sezer et al., (2019) [19]	Halogen, xenon, LED at equal lumen output	1.5 cm distance, 30 and 60 min	Halogen caused significant DNA damage, elevated TNF-α, IL-1β, IL-6, and increased apoptosis at 60 min; xenon and LED showed no significant differences from controls
Yanagi et al., (2006) [21]	20G and 25G endoilluminators (halogen, metal halide)	1 cm distance, 30 min	Two of three 20G endoilluminators caused significant cell death; 25G endoilluminators did not affect viability
Yanagi et al., (2007) [22]	Xenon/bandpass vs. halogen	1–2 cm distance, 5–30 min	Comparable A2E-mediated phototoxicity at equivalent luminous flux; chandelier probe induced damage similar to 20G halogen
Balaiya et al., (2014) [20]	Metal halide with BBG dye	1 cm and 2.5 cm, 1–15 min	At 1 cm, viability dropped to 38.9% after 15 min with 0.25 mg/mL BBG; at 2.5 cm, viability was 62.07% at 15 min

#### Reduced endoillumination enabled by digital visualization

Two studies directly examined whether reduced endoillumination levels compromise surgical outcomes. Ge et al. (2025) [24], in the only RCT identified, demonstrated that 3D digital visualization system allowed completion of pars plana vitrectomy for rhegmatogenous retinal detachment with optical fiber and chandelier settings at 15% of maximum output, thus significantly lower than the 43–47% (fibers) and 32–36% (chandeliers) required for traditional eyepiece surgery (*p* < 0.001). At six months, retinal reattachment was 100% in both groups, with no significant differences in best-corrected visual acuity, intraocular pressure, or flash electroretinogram.

Adam et al. (2017) [25] found that surgeons could operate comfortably at 10% of maximum endoillumination output using a 3D digital visualization system, with corresponding display luminous emittance of 14.3 ± 9.5 lx (measured 40 cm from the display), with one surgeon comfortable at as low as 3%. No intraoperative complications occurred. These findings indicate that digital image amplification can may reduce the required illumination output by approximately 70–90% without compromising surgical performance.

#### Instrument design and gauge effects

The evolution toward smaller-gauge systems (25G, 27G) has had direct safety implications beyond wound construction. Oshima et al. (2010) [26] developed a 27/29G illumination light pipe that provided sufficient wide-angle illumination comparable to chandelier systems while benefiting from improved rigidity and single-step entry. The 27G chandelier endoilluminator reported by Oshima et al. (2007) [28] provided up to 25 lm, brighter than commercially available light pipe-style endoilluminators at 5–15 lm, while its self-retaining, sutureless design allowed bimanual manipulation.

#### Role of spectral filters

Table 6 summarizes the main findings on the role of spectral filters and their impact on safety and visualization. Coppola et al. (2017) [13] concluded that amber filters showed superiority over green and yellow filters for reducing phototoxicity, particularly in dye-assisted chromovitrectomy. However, the authors noted that histological donor eye studies and large multicenter trials are still needed to validate the degree of photoprotection conferred by spectral filters.

**Table 6:** Role of spectral filters

Filter type	Mechanism	Impact on safety	Impact on visualization
420 nm cutoff	Blocks UV and deep blue [22]	Moderate improvement in threshold time [3]	Minimal color distortion [5]
435 nm cutoff	Blocks UV through violet-blue [5]	Increases threshold time ~ 60% vs. 420 nm filter [3]	Slight yellow tint [5]
475–485 nm cutoff	Blocks through mid-blue [8]	Extends safe exposure substantially (up to 13 min at 10 mm) [8]	Noticeable yellow shift [3]
515 nm cutoff	Blocks blue-green [13]	Maximum photochemical protection [13]	Strong yellow hue; reduced color contrast [18]
Amber filter	Broad short-wavelength attenuation [13]	Superior to green and yellow filters [13]	Reduces luminance but does not alter color contrast [13]

## Discussion

### Established endoillumination systems

The safety of established endoillumination systems cannot be interpreted solely according to the nominal light-source category, such as halogen, xenon, mercury vapor, or LED. Across the studies included in this review, retinal light exposure depends on the interaction between spectral emission profile, probe-to-retina distance, delivery geometry, spectral filtering, and visualization platform. Therefore, comparisons between systems are most meaningful when these variables are considered together rather than when technologies are treated as homogeneous classes. This is particularly evident for LED-based endoillumination. Fehler et al. (2023) [6] found that the DORC LEDStar had the shortest maximum exposure times among the three source types tested, with only 2 min 9 s at 2 mm compared to 11 min 13 s for halogen, suggesting a higher retinal risk. Conversely, Dithmar et al. (2011) [7] reported that the Geuder ocuLED had a blue light proportion of only 15.5–22.3%, lower than xenon (28.8–30.8%), and classified it as safe from phototoxic damage. The in vitro RPE cell study similarly found LED light comparable to controls and safer than halogen at equal lumen output [19]. These apparently conflicting findings reflect the substantial variability of LED spectra across manufacturers and color temperatures: the DORC LEDStar shows a pronounced 440 nm emission peak [6], whereas the Geuder ocuLED neutral white has a lower-intensity 455 nm secondary peak [7]. LED safety should therefore be assessed at the level of the individual device rather than as a class effect. Among conventional high-output sources, mercury vapor appears to offer a favorable safety-efficacy profile, combining high luminous efficacy (402 lm/W) [13], long threshold times at clinically relevant distances (15 min 16 s at 4 mm; 50 min 59 s at 8 mm) [3], and a relatively low blue-light proportion among non-LED sources (14.1%) [7]. However, the practical relevance of source-type comparisons is increasingly limited by system-level factors, particularly light delivery geometry, filtering, and image amplification.

The most consistent safety advantage across the included studies was associated not with any particular light source type, but with chandelier delivery geometry. By fixing the light source 8–16.5 mm from the retina, compared with the typical 2–5 mm distance of handheld probes, chandeliers exploit the inverse-square law to reduce retinal irradiance by an order of magnitude [3, 23]. The Synergetics 25G chandelier achieved a threshold time of over 4 h at maximum illumination [3], whereas handheld probes often reached comparable thresholds within minutes. Chandelier systems also support bimanual surgery and reduce the need for continuous manual probe direction, thereby limiting the risk of inadvertent close-proximity retinal exposure [4, 5].

However, these advantages must be balanced against optical and technical limitations. Because chandelier systems predominantly provide fixed, diffused illumination, they may offer lower contrast on transparent structures than a movable focal probe [1, 2] Surgical instrument may also interrupt the light path creating shadows [2–4], while reposition may be required to achieve compete peripheral visualization [3, 4]. Additional concerns include the need of additional sclerotomy, and the possible wound leakage [2–4, 28].

Beyond these chandelier-specific limitations, intraocular irradiance may further complicate safety estimates, as measured intraocular values exceeded bench-top reference measurements by 30–60% [12]. This effect was observed independently of iris pigmentation [12], suggesting that it is primarily related to intraocular geometry rather than pigment-dependent absorption. Published safety margins should therefore be interpreted conservatively across all technologies.

Three-dimensional digital visualization platforms are less directly related to intrinsic endoillumination safety, but they can reduce cumulative retinal light exposure by lowering the illumination intensity required for surgery. By digitally amplifying the surgical image, these platforms can allow clinically effective visualization at 10–15% of conventional illumination intensity [24, 25]. Ge et al.’s (2025) randomized controlled trial [24] provides the strongest evidence within this review for such an illumination-reduction strategy. When combined with chandelier delivery and appropriate spectral filtering, 3D visualization may therefore contribute to a broader system-level approach to minimizing retinal light exposure, shifting the discussion from the safety of individual light sources to the optimization of the entire visualization and illumination setup.

### Alternative and emerging illumination systems

In addition to incremental improvements in conventional fiber-optic endoillumination, several alternative strategies have been developed to address limitations of traditional systems, including transmission inefficiency, limited flexibility, and retinal phototoxicity. These approaches aim to improve light delivery efficiency, simplify surgical workflow, and enhance safety through optimized geometry and spectral control.

A central line of innovation involves miniaturized LED-based systems designed to reduce or eliminate dependence on external light sources and optical fibers. Conventional small-gauge fiber systems are inherently inefficient, delivering only a small fraction of the generated intraocularly, which necessitates high-power light sources [29]. To overcome this limitation, prototype devices integrate the LED directly at or near the emission site, significantly reducing transmission loss and simplifying system architecture. Experimental studies in porcine eyes demonstrated that such miniature LED systems can provide adequate intraocular illumination with substantially lower total luminous flux, while maintaining photochemical and thermal exposure well below safety limits [29].

One variant of this approach is the intraocular micro-LED “chip-on-tip” system, in which the light source is positioned at the probe tip. This design enables homogeneous illumination, physiologic color rendering, and prolonged safe exposure times (up to several hours under defined conditions), while eliminating bulky external light sources [30]. However, because irradiance still increases sharply at short working distances, safety remains dependent on probe positioning.

An alternative strategy is extraocular transscleral illumination, in which a white LED is applied externally to the sclera. This approach avoids insertion of an additional intraocular illumination probe; however, its clinical applicability is likely limited to selected peripheral maneuvers. Experimental data in porcine models showed that a complete intraocular illumination can be achieved via scleral transmission, although with reduced brightness and a red-shifted spectrum due to wavelength-dependent filtering by the sclera [29, 31]. Importantly, this indirect illumination pathway substantially lowers retinal irradiance and allows markedly longer theoretical exposure times, improving safety. However, the diffuse and non-directional nature of the light may limit its use in procedures requiring precise focal illumination.

Optimization of illumination geometry has also led to omnidirectional diffuser-based systems, such as the LED ORB platform. By incorporating a diffuser at the fiber tip, these systems achieve near-360° illumination of the vitreous cavity, enabling uniform fundus visualization with a single fiber and reducing the need for repositioning or multiple light sources [32]. This configuration is particularly advantageous for wide-angle viewing systems and bimanual surgery, although it does not eliminate fiber-related transmission losses.

Finally, portable LED-based devices have been proposed as supplemental or emergency illumination tools. The Retilight, a 3D-printed portable LED system, can be connected to standard endoillumination fibers and operated independently of the main surgical console [33]. Despite lower illuminance compared with conventional systems, it provides sufficient illumination for surgical use and may be particularly valuable in cases of equipment failure or in resource-limited settings.

Overall, emerging illumination technologies reflect a consistent trend toward fiber-free or fiber-optimized designs, improved energy efficiency, and safer illumination profiles. While many of these systems remain experimental, they highlight a shift from high-power centralized light sources toward localized, controllable, and potentially less hazardous illumination strategies in vitreoretinal surgery.

### Limitations of this work

This review has some limitations that should be considered when interpreting its findings. First, the marked heterogeneity of the available literature - comprising in vitro cell-culture work, ex vivo porcine-eye measurements, optical bench tests, clinical case series, and a single randomized controlled trial, with no common quantitative outcome - made a systematic review with meta-analysis unfeasible and precluded any pooled quantitative synthesis or direct comparison across studies. A narrative approach was therefore adopted, and no formal quantitative risk-of-bias assessment was performed; the critical appraisal was consequently qualitative and inherently subject to reviewer judgment. Second, much of the safety evidence derives from ex vivo porcine-eye models and retinal pigment epithelium cultures, which may not fully reproduce the optical, anatomical, vascular, and metabolic conditions of the living human eye, so absolute exposure thresholds should be regarded as approximate rather than definitive. Third, the available clinical evidence is constrained by small sample sizes, heterogeneous surgical indications, short follow-up, and the identification of only one randomized controlled trial, limiting the strength of conclusions regarding clinical safety and efficacy. Finally, spectral output, irradiance, and calculated exposure-time data were strongly device- and manufacturer-specific, and results obtained with a given light source or probe cannot be generalized across the broader device category. These limitations should be weighed when translating the present findings into clinical practice.

## Supplementary Information

Below is the link to the electronic supplementary material.

Supplementary Material 1

Supplementary Material 2

## Data Availability

No datasets were generated or analysed during the current study.
