# Supplementary material for: Illumination systems in vitreoretinal surgery: a comparative review of safety and efficacy
Source: Int J Retina Vitreous. 2026 Aug 5;12:150. doi: 10.1186/s40942-026-00918-y (PMC13732959; doi:10.1186/s40942-026-00918-y)
Supplement: Supplementary file 1 [file 40942_2026_918_MOESM2_ESM.pdf]

Article	Study Type	Model	Light Source	System	Sample	Main Findings	Main appraisal strengths	Main limitations	Overall appraisal concern
Chow, 2011 [3]	Review	Literature and clinical safety concepts in vitreoretinal illumination	Halogen; xenon; mercury vapour	Clinical light sources and filters used in vitreoretinal surgery	Not applicable	The article emphasises that retinal threshold time is more clinically meaningful than aphakic hazard sum alone, and that increasing working distance is one of the strongest ways to improve safety; in the safety hierarchy summarised by the author, mercury vapour performed best, followed by 435-nm–filtered xenon, then halogen.	Practical surgical relevance: clearly explains the effect of brightness, filtering, and working distance	No original data; lower evidentiary weight	High
Sakaguchi & Oshima, 2012 [4]	Review	Modern vitreoretinal illumination choices for small-gauge surgery	Halogen; metal halide; xenon; mercury vapour	Small-gauge light probes, wide-angle probes, chandelier systems, filtered illuminators	Not applicable	The review concludes that xenon and mercury vapour sources overcame the brightness limitations of small-gauge halogen systems; it also argues that chandelier illumination is theoretically safest because greater working distance markedly prolongs retinal threshold time, with reported threshold times increasing substantially when distance is doubled.	Broad clinically oriented overview of xenon, mercury vapour, filters, chandelier illumination and small-gauge surgery; useful contextual synthesis	Narrative selection; includes personal experience and industry literature; no original data	High
Chow, 2014 [5]	Review	Literature on vitreoretinal light sources and endoillumination evolution	Halogen; metal halide; xenon; mercury vapour; LED	Conventional light pipes, wide-angle probes, chandeliers, and filtered platforms	Not applicable	The review describes the transition from underpowered halogen/metal halide systems to xenon, mercury vapour, and newer LED platforms; based on comparative safety calculations, mercury vapour ranked safest, followed by xenon with 435-nm filtering, then halogen, whereas older xenon/metal halide configurations carried higher hazard.	Comprehensive historical and technical overview; explains aphakic hazard sum, filters, working distance, and threshold time	Expert narrative source; not original clinical research; potential selection bias	High
Sadeghi et al., 2023 [12]	Review	Recent imaging and surgical visualisation technologies in vitreoretinal surgery	Halogen; metal halide; xenon; mercury vapour; LED; laser-light systems	Endoillumination devices, chandeliers, filters, wide-angle systems, 3D platforms	Not applicable	The review summarises the shift from older halogen/metal halide sources to brighter xenon, mercury vapour, and LED systems, highlights the broad utility of chandelier illumination for bimanual surgery, and notes that improved visualisation technologies may help reduce retinal phototoxicity by lowering illumination requirements.	Recent overview of surgical visualisation technologies, including endoillumination, filters, chromovitrectomy, and 3D systems	Broad scope; not focused specifically on endoillumination; narrative selection strategy	Moderate–high
Coppola et al., 2017 [13]	Review	Literature on filters and endoillumination safety	Multiple light sources with spectral filters	Spectral pass filters in modern vitreoretinal surgery	Not applicable	Spectral filters were reported to significantly reduce phototoxic risk and extend allowable exposure times; amber filters appeared more favourable than green/yellow filters for some visualisation tasks, and filter use was strongly recommended, especially in dye-assisted chromovitrectomy.	Useful synthesis on filters, chromovitrectomy, phototoxicity mechanisms; clinically relevant discussion of spectral filtering	Narrative/non-systematic; no original measurements; limited objective appraisal of included studies	Moderate
Fehler et al., 2023 [6]	Experimental ex vivo comparative study	Porcine eyes, foveal irradiance measurements	Halogen; xenon; LED	Handheld and chandelier illumination systems at varying distances	Ex vivo porcine-eye comparison across multiple source/fibre combinations	At maximum brightness, retinal hazard was lowest for halogen, intermediate for xenon, and highest for LED; with brightness normalisation, xenon and LED showed similar hazard while halogen remained safer. The authors advise avoiding maximum intensity, especially with xenon and LED systems.	Strong technical methodology; compares halogen, xenon and LED; evaluates distance, fibre type, photochemical/thermal hazard and maximum exposure time according to standards	Ex vivo model; no biological retinal damage endpoint; clinical extrapolation required	Low–moderate
Sezer et al., 2018 [19]	Experimental in vitro comparative study	ARPE-19 human RPE cell cultures	Halogen; xenon; LED	Endoillumination light sources used at equal lumen output	ARPE-19 cultures	At equal lumen output, halogen caused significantly greater DNA damage, inflammatory cytokine release, reduced viability, and more apoptosis than controls, whereas xenon and LED showed no significant toxicity under the tested conditions.	Compares three clinically relevant light sources at equal lumen output; evaluates viability, DNA damage, apoptosis and inflammatory markers	Cell culture model only; LED source was non-commercial; exposure conditions may not fully reproduce real damage	Moderate
Koelbl et al., 2019 [12]	Experimental ex vivo study	Porcine eyes with spotlight and wide-angle endoilluminators	Halogen	23G spotlight and 23G wide-angle endoilluminators	14 porcine eyes (9 brown irises, 5 blue irises)	Intraocular retinal irradiance was consistently 30–60% higher than the safe guide (10 J/cm2) with the same setup; spotlight illumination generated higher local irradiance than wide-angle illumination, and iris pigmentation had no significant effect. The study suggests that currently accepted “safe” exposure times may be overestimated.	Strong translational relevance; compares reference vs intraocular irradiance; shows intraocular irradiance may be 30–60% higher	Ex vivo animal model; limited light-source types; does not measure biological tissue damage	Low–moderate
Balaiya et al., 2014 [20]	Experimental in vitro study	ARPE-19 human RPE cells exposed to Brilliant Blue Green (BBG) under endoillumination	Metal halide	Surgical endoilluminator in chromovitrectomy model with BBG	ARPE-19 cultures	BBG toxicity increased with longer exposure and shorter illumination distance; at the clinically used concentration (0.25 mg/mL), BBG was considered safe for up to 5 min at 1 cm and 15 min at 2.5 cm, providing practical safety parameters for dye-assisted surgery.	Clinically relevant assessment of BBG concentration, illumination distance and exposure duration; includes viability and morphometric analysis	Focuses on dye-light interaction rather than the illumination system alone; in vitro model; limited to a metal halide light source	Moderate
Yanagi et al., 2006 [21]	Experimental in vitro study + theoretical hazard analysis	A2E-laden RPE cells; 20G and 25G endoilluminators	Halogen; metal halide (“halogen-like” and “xenon-like” outputs)	Six commercially available endoilluminators from two manufacturers	ARPE-19 culture	Theoretical retinal threshold times were <1 min for all devices; significant A2E-mediated viability loss was seen with two of three 20G probes, whereas 25G probes did not reduce viability under the same conditions; theoretical hazard estimates did not correlate with experimental toxicity.	Strong biological relevance for blue-light/RPE toxicity; combines spectral irradiance with cell viability; compares 20G and 25G systems	The A2E-cell model does not fully reproduce clinical surgery; theoretical hazard times did not correlate well with experimental data	Moderate
Yanagi et al., 2007 [22]	Experimental in vitro study	A2E-laden RPE cells exposed to xenon/bandpass vs halogen illumination	Xenon/bandpass; halogen	20G and 25G xenon endoilluminators; chandelier probe; 20G and 25G halogen comparator	ARPE-19 culture	Xenon/bandpass light through a 20G probe had 2.8-fold higher luminous flux than halogen, yet when total luminous flux was matched, A2E-mediated phototoxicity was comparable to halogen source; chandelier xenon/bandpass illumination induced damage similar to 20G halogen.	Strong experimental design for photochemical toxicity; compares xenon/bandpass with halogen; evaluates standard and chandelier probes; uses luminous flux and spectral irradiance measurements	An in vitro model cannot fully reproduce intraocular conditions; short-term cell viability endpoint only; no clinical outcome correlation	Moderate
Koch et al., 1993 [23]	Experimental bench / physical optics study	Multiport illumination system vs conventional fibreoptic light pipes	Halogen	Pars plana–fixated multiport illumination system, with 3 fibreoptic cables	No formal sample	Although retinal irradiance at 5 mm for conventional handheld systems ranged from roughly 94 to 256 mW/cm², the actual pars plana–fixated multiport configuration delivered substantially lower retinal irradiance because of the longer working distance; reducing intensity further lowered irradiance, and a filter markedly reduced short-wavelength output.	Direct irradiance and spectral measurements; clinically relevant distances; compares multiport illumination with conventional light pipes	Older technology; limited biological/clinical outcome validation	Moderate-high

Ge et al., 2024 [24]	Prospective randomised controlled trial	Human eyes undergoing Pars plana vitrectomy (PPV) for rhegmatogenous retinal detachment	Endoillumination system with optical fibre and chandelier (same wavelength source; different intensity settings between groups)	3D low-light intensity-assisted PPV versus traditional eyepiece-assisted PPV	40 eyes of 40 patients (20 in the 3D group, 20 in the eyepiece group)	PPV was successfully completed in all eyes, with no significant difference in surgery time between groups. The 3D group used significantly lower illumination intensity than the eyepiece group for both optical fibres and chandeliers at 5 mm and 1 cm (all $p < 0.001$). At 6 months, both groups achieved 100% retinal attachment, with no significant differences in Best-Corrected Visual Acuity, Intraocular Pressure, Full-field Electroretinography, or complication rates. The study concluded that 3D low-light PPV achieved equivalent anatomical and functional outcomes while reducing intraoperative light intensity and theoretically lowering retinal light injury risk.	Strong clinical design; randomised comparison; quantifies intraoperative light intensity; 6-month follow-up; includes anatomical and functional outcomes	Small sample size; single-centre; limited to RRD; not primarily designed to detect phototoxicity events	Low–moderate
Adam et al., 2016 [25]	Prospective observational case series	Human vitreoretinal surgery using a 3D heads-up display	Xenon (Constellation platform)	3D HUD vitreoretinal surgery with light pipe/chandelier illumination	10 eyes (10 patients)	In 9/10 cases, surgeons were comfortable at 10% endoillumination (mean HUD emittance 14.3 ± 9.5 lux); in the remaining case, 3% was acceptable. Lower levels caused dimness/noise. The findings support substantially reduced illumination requirements and a theoretical reduction in phototoxic risk.	Clinically relevant human surgical setting; measured HUD luminous emittance; directly evaluates reduced illumination in 3D surgery	Very small sample; subjective surgeon-defined visibility threshold; no long-term retinal toxicity assessment	Moderate
Oshima et al., 2010 [26]	Experimental interventional case series with bench testing	Human 27G Microincision Vitrectomy Surgery plus in vitro fluidics/cutter testing	Xenon; mercury vapour	27G transconjunctival vitrectomy system, including illumination, cutter, infusion, and instruments	31 eyes (31 patients)	The 27G system was feasible and clinically effective: anatomic success was 100%, visual improvement of≥3 lines occurred in 65%, and all sclerotomies self-sealed without hypotony. The 27/29G light pipe produced 20 lumens with xenon and 25 lumens with mercury vapour, providing sufficient wide-angle illumination for intraocular manoeuvres.	Combines bench testing, instrument development, and clinical feasibility; reports anatomic/visual outcomes and complications	Small uncontrolled pilot series; illumination is one component of a broader instrument system	Moderate
Horiguchi et al., 2002 [27]	Technical report / interventional case series	Human eyes undergoing bimanual vitreoretinal surgery	50W halogen microscope light	Fiberoptic-free bimanual vitreous surgery system using a 40 Diopter aspheric lens + prismatic inverter	37 eyes (37 patients)	The microscope-based non-fibreoptic system was used successfully in all cases; 30/37 eyes improved by ≥2 lines, and no phototoxicity was reported, supporting the feasibility for bimanual surgery without intraocular fibreoptic illumination.	Human surgical experience; reports visual outcomes and absence of observed phototoxicity	No control group; phototoxicity assessed clinically only; device-specific applicability	Moderate
Dithmar et al., 2011 [7]	Bench comparative technical evaluation + first intraoperative use	Prototype LED system compared with commercial vitreoretinal light sources	LED; xenon; mercury vapour	Handle-integrated ocuLED system vs conventional external-source systems	No formal sample	The LED-based system showed a lower blue-light proportion than xenon, maximum radiant power/irradiance in the range of xenon and mercury vapour systems, and good intraoperative visibility; the authors concluded that LED technology was sufficient and safe for vitreoretinal illumination.	Detailed spectral, radiant power, and irradiance measurements; comparison with commercial xenon/mercury systems; includes surgical handling assessment	Prototype device; limited clinical data; no biological toxicity model	Moderate
van den Biesen et al., 2000 [8]	Experimental bench safety study	Commercial vitreoretinal illumination systems evaluated against International Commission on Non-Ionising Radiation Protection (ICNIRP) safety guidelines	Halogen; metal halide; xenon	Standard 300µm 20G fibreoptic endoillumination probe with multiple filter combinations	No formal sample	Photochemical safety limits were exceeded within 1 min in 9/10 combinations; using a 475-nm long-pass filter, lowering power, and increasing probe-retina distance increased the safe-exposure window, in some conditions up to 13 min; thermal damage was predicted when the probe touched the retina.	Strong physical characterisation; compares multiple light-source/filter combinations; provides exposure-time estimates	Theoretical model; older devices; clinical correlation limited	Moderate
Oshima et al., 2007 [28]	New instrument / clinical technical report	Vitreoretinal surgical use in human eyes	Xenon	Self-retaining 27G chandelier endoilluminator for panoramic viewing	Variety of surgical cases; number not reported	The 27G chandelier provided up to 25 lumens, enabled hands-free wide-angle illumination, avoided glare and sutures, and was reported to improve surgical efficiency and efficacy without associated surgical complications in the described use.	Clear device description; clinically relevant for bimanual surgery; reports wide-angle illumination and sutureless use	Brief report; limited quantitative safety data; no comparator group	Moderate–high
Hessling et al., 2015 [29]	New instrument / clinical technical report	Porcine eyes; two LED-based illumination concepts	White LED	Miniature LED chandelier endoilluminator and transscleral LED endoilluminator	Porcine eyes; number not specified	The paper describes two fibre-free LED concepts. The miniature LED chandelier showed a calculated maximum safe exposure time of ~13 h, whereas the transscleral LED system showed ~44 h; both provided sufficient brightness in porcine eyes. The transscleral system appeared dimmer and more red-shifted, but both approaches were presented as potentially safer, cheaper, and more convenient than conventional xenon/fibre systems.	Evaluates two novel LED approaches; includes photochemical/thermal hazard calculations and porcine-eye feasibility testing	Prototype/preclinical study; limited quantitative clinical validation; no human surgical outcomes	Moderate
Koelbl et al., 2019 [30]	New instrument / clinical technical report	Porcine eyes; intraocular micro-LED endo-illumination	micro-LED	Intraocular micro LED light-source-on-tip endo-illuminator (23G-compatible)	10 porcine eyes	The intraocular micro-LED device generated homogeneous, bright, physiologic illumination and combined chandelier-style wide-angle illumination with a navigable spot. Despite the intraocular light source, calculated retinal hazard remained low, with a continuous safe exposure time of at least 4.7 h even at 0 mm distance; thermal values were far below ISO limits.	Detailed light-source-on-tip concept; evaluates photochemical and thermal hazards according to standards; reports potential continuous exposure time ≥4.7 h	Preclinical porcine-eye model; no human data; device feasibility more than clinical effectiveness	Moderate
Kölbl et al., 2015 [31]	New instrument / clinical technical report	Porcine eyes; transscleral extraocular illumination model	White LED	Extraocular non-invasive transscleral LED endoilluminator integrated into an eye speculum.	Porcine eyes; number not specified	A speculum-mounted transscleral LED provided acceptable and uniform intraocular illumination without an incision. Safety calculations showed very low retinal hazard, with a calculated photochemical exposure time of ~44 h at 18 mm and ~120 min at 1 mm; measured surface temperature remained below damaging levels. The authors concluded that the approach could markedly reduce phototoxic stress compared with conventional systems.	Innovative non-invasive illumination concept; evaluates photochemical and thermal safety according to standards; includes porcine-eye feasibility testing	Prototype stage; no human clinical validation; limited surgical performance data	Moderate
Torii et al., 2021 [32]	New instrument / clinical technical report	Intraoperative vitreoretinal surgery chendelier	LED	LED ORB Light System, an omnidirectional 360° chandelier fibre illumination device	No formal sample	The ORB system used a single fibre with a diffuser tip to achieve 360° fundus illumination, overcoming the limited illumination angle of conventional chandelier fibres. Reported illuminance was ~6,500 lx with the 25G fibre, and ~4,000 lx with the 27G fibre, and retinal phototoxicity limits were reportedly not reached until ~3 h of vitreous surgery. The device enabled even illumination, easier manoeuvring, and improved endoscopic viewing.	Clinically relevant 360° illumination concept; reports adequate illumination with a single fibre and estimated phototoxicity threshold	Device report; limited quantitative safety data; no controlled clinical comparison	Moderate–high

Chandrakanth et al., 2025 [33]	New instrument / clinical technical report	Portable backup or supplemental illumination for vitreoretinal surgery	LED	Retilight, a 3D-printed portable LED endo-illuminator compatible with existing optical fibre cables	No formal sample	Retilight was developed as a portable, low-cost backup or supplemental LED light source for vitreoretinal surgery. External lux testing showed 10,500 lux for Retilight versus 15,100 lux for the Alcon halogen source and 15,800 lux for a standard external LED source. The reported total prototype cost was 5,000 Indian rupees, and the device was proposed as a practical option during power outages, bulb failure, or chandelier-assisted bimanual surgery.	Practical low-cost portable LED backup system; includes prototype development and lux comparison with existing systems.	Early innovation report; limited safety testing; no formal intraocular phototoxicity assessment; no comparative clinical outcomes	High
